# Meta-Analysis of Mismatch Repair Polymorphisms within the Cogent Consortium for Colorectal Cancer Susceptibility

**DOI:** 10.1371/journal.pone.0072091

**Published:** 2013-09-06

**Authors:** Simone Picelli, Justo Lorenzo Bermejo, Jenny Chang-Claude, Michael Hoffmeister, Ceres Fernández-Rozadilla, Angel Carracedo, Antoni Castells, Sergi Castellví-Bel, Members of the EPICOLON Consortium (Gastrointestinal Oncology Group of the Spanish Gastroenterological Association):, Diego Morillas Juan, Muñoz Raquel, Manzano Marisa, Colina Francisco, Díaz Jose, Ibarrola Carolina, López Guadalupe, Ibáñez Alberto, Castells Antoni, Piñol Virgínia, Castellví-Bel Sergi, Balaguer Francesc, Gonzalo Victoria, Ocaña Teresa, Giráldez María Dolores, Pellisé Maria, Serradesanferm Anna, Moreira Leticia, Cuatrecasas Miriam, M. Piqué Josep, Lanas Ángel, Alcedo Javier, Ortego Javier, Cubiella Joaquin, Soledad Díez Ma, Salgado Mercedes, Sánchez Eloy, Vega Mariano, Andreu Montserrat, Abuli Anna, Bessa Xavier, Iglesias Mar, Seoane Agustín, Bory Felipe, Navarro Gemma, Bellosillo Beatriz, Ma Dedeu Josep, Álvarez Cristina, Puigvehí Marc, Bujanda Luis, Cosme Ángel, Gil Inés, Larzabal Mikel, Placer Carlos, del Mar Ramírez María, Hijona Elisabeth, M. Enríquez-Navascués Jose, L. Elosegui Jose, Payá Artemio, Jover Rodrigo, Alenda Cristina, Sempere Laura, Acame Nuria, Rojas Estefanía, Pérez-Carbonell Lucía, Rigau Joaquim, Serrano Ángel, Giménez Anna, Saló Joan, Batiste-Alentorn Eduard, Autonell Josefina, Barniol Ramon, María García Ana, Carballo Fernando, Bienvenido Antonio, Sanz Eduardo, González Fernando, Sánchez Jaime, Ono Akiko, Latorre Mercedes, Medina Enrique, Cuquerella Jaime, Canelles Pilar, Martorell Miguel, Ángel García José, Quiles Francisco, Orti Elisa, Clofent Juan, Seoane Jaime, Tardío Antoni, Sanchez Eugenia, Luisa de Castro Ma, Tardío Antoni, Clofent Juan, Hernández Vicent, Llor Xavier, M. Xicola Rosa, Piñol Marta, Rosinach Mercè, Roca Anna, Pons Elisenda, M. Hernández José, A. Gassull Miquel, Fernández-Bañares Fernando, M. Viver Josep, Salas Antonio, Espinós Jorge, Forné Montserrat, Esteve Maria, M. Reñé Josep, Piñol Carmen, Buenestado Juan, Viñas Joan, Quintero Enrique, Nicolás David, Parra Adolfo, Martín Antonio, Argüello Lidia, Pons Vicente, Pertejo Virginia, Sala Teresa, Gonzalez Dolors, Roman Eva, Ramon Teresa, Poca Maria, Mar Concepción Ma, Martin Marta, Pétriz Lourdes, Martinez Daniel, Carracedo Ángel, Ruiz-Ponte Clara, Fernández-Rozadilla Ceres, Magdalena Castro Ma, Riestra Sabino, Rodrigo Luis, Fernández Javier, Luis Cabriada Jose, Carreño Luis, Oquiñena Susana, Bolado Federico, Peña Elena, Manuel Blas José, Ceña Gloria, José Sebastián Juan, Naranjo Antonio, Alessio Naccarati, Barbara Pardini, Ludmila Vodickova, Heiko Müller, Bente A. Talseth-Palmer, Geoffrey Stibbard, Paolo Peterlongo, Carmela Nici, Silvia Veneroni, Li Li, Graham Casey, Albert Tenesa, Susan M. Farrington, Ian Tomlinson, Victor Moreno, Tom van Wezel, Juul Wijnen, Malcolm Dunlop, Paolo Radice, Rodney J. Scott, Pavel Vodicka, Clara Ruiz-Ponte, Hermann Brenner, Stephan Buch, Henry Völzke, Jochen Hampe, Clemens Schafmayer, Annika Lindblom

**Affiliations:** Hospital 12 de Octubre, Madrid; Hospital 12 de Octubre, Madrid; Hospital 12 de Octubre, Madrid; Hospital 12 de Octubre, Madrid; Hospital 12 de Octubre, Madrid; Hospital 12 de Octubre, Madrid; Hospital 12 de Octubre, Madrid; Hospital 12 de Octubre, Madrid; Alberto Ibáñez; Hospital Clínic, Barcelona; Alberto Ibáñez; Hospital Clínic, Barcelona; Alberto Ibáñez; Hospital Clínic, Barcelona; Alberto Ibáñez; Hospital Clínic, Barcelona; Alberto Ibáñez; Hospital Clínic, Barcelona; Alberto Ibáñez; Hospital Clínic, Barcelona; Alberto Ibáñez; Hospital Clínic, Barcelona; Alberto Ibáñez; Hospital Clínic, Barcelona; Alberto Ibáñez; Hospital Clínic, Barcelona; Alberto Ibáñez; Hospital Clínic, Barcelona; Alberto Ibáñez; Hospital Clínic, Barcelona; Alberto Ibáñez; Hospital Clínic, Barcelona; Hospital Clínico Universitario, Zaragoza; Hospital Clínico Universitario, Zaragoza; Hospital Clínico Universitario, Zaragoza; Hospital Cristal-Piñor, Complexo Hospitalario de Ourense; Hospital Cristal-Piñor, Complexo Hospitalario de Ourense; Hospital Cristal-Piñor, Complexo Hospitalario de Ourense; Hospital Cristal-Piñor, Complexo Hospitalario de Ourense; Hospital Cristal-Piñor, Complexo Hospitalario de Ourense; Parc de Salut Mar, Barcelona; Parc de Salut Mar, Barcelona; Parc de Salut Mar, Barcelona; Parc de Salut Mar, Barcelona; Parc de Salut Mar, Barcelona; Parc de Salut Mar, Barcelona; Parc de Salut Mar, Barcelona; Parc de Salut Mar, Barcelona; Parc de Salut Mar, Barcelona; Parc de Salut Mar, Barcelona; Parc de Salut Mar, Barcelona; Hospital San Eloy, Baracaldo and Hospital Donostia, CIBERehd, University of Basque Country, San Sebastián; Hospital San Eloy, Baracaldo and Hospital Donostia, CIBERehd, University of Basque Country, San Sebastián; Hospital San Eloy, Baracaldo and Hospital Donostia, CIBERehd, University of Basque Country, San Sebastián; Hospital San Eloy, Baracaldo and Hospital Donostia, CIBERehd, University of Basque Country, San Sebastián; Hospital San Eloy, Baracaldo and Hospital Donostia, CIBERehd, University of Basque Country, San Sebastián; Hospital San Eloy, Baracaldo and Hospital Donostia, CIBERehd, University of Basque Country, San Sebastián; Hospital San Eloy, Baracaldo and Hospital Donostia, CIBERehd, University of Basque Country, San Sebastián; Hospital San Eloy, Baracaldo and Hospital Donostia, CIBERehd, University of Basque Country, San Sebastián; Hospital San Eloy, Baracaldo and Hospital Donostia, CIBERehd, University of Basque Country, San Sebastián; Hospital General Universitario de Alicante; Hospital General Universitario de Alicante; Hospital General Universitario de Alicante; Hospital General Universitario de Alicante; Hospital General Universitario de Alicante; Hospital General Universitario de Alicante; Hospital General Universitario de Alicante; Hospital General de Granollers; Hospital General de Granollers; Hospital General de Granollers; Hospital General de Vic; Hospital General de Vic; Hospital General de Vic; Hospital General de Vic; Hospital General Universitario de Guadalajara and Fundación para la Formación e Investigación Sanitarias Murcia; Hospital General Universitario de Guadalajara and Fundación para la Formación e Investigación Sanitarias Murcia; Hospital General Universitario de Guadalajara and Fundación para la Formación e Investigación Sanitarias Murcia; Hospital General Universitario de Guadalajara and Fundación para la Formación e Investigación Sanitarias Murcia; Hospital General Universitario de Guadalajara and Fundación para la Formación e Investigación Sanitarias Murcia; Hospital General Universitario de Guadalajara and Fundación para la Formación e Investigación Sanitarias Murcia; Hospital General Universitario de Guadalajara and Fundación para la Formación e Investigación Sanitarias Murcia; Hospital General Universitario de Valencia; Hospital General Universitario de Valencia; Hospital General Universitario de Valencia; Hospital General Universitario de Valencia; Hospital General Universitario de Valencia; Hospital General Universitario de Valencia; Hospital General Universitario de Valencia; Hospital General Universitario de Valencia; CHUVI-Hospital Meixoeiro, Vigo: EPICOLON I; CHUVI-Hospital Meixoeiro, Vigo: EPICOLON I; CHUVI-Hospital Meixoeiro, Vigo: EPICOLON I; CHUVI-Hospital Meixoeiro, Vigo: EPICOLON I; EPICOLON II; EPICOLON II; EPICOLON II; EPICOLON II; Hospital Universitari Germans Trias i Pujol, Badalona and Section of Digestive Diseases and Nutrition, University of Illinois at Chicago, IL, USA; Hospital Universitari Germans Trias i Pujol, Badalona and Section of Digestive Diseases and Nutrition, University of Illinois at Chicago, IL, USA; Hospital Universitari Germans Trias i Pujol, Badalona and Section of Digestive Diseases and Nutrition, University of Illinois at Chicago, IL, USA; Hospital Universitari Germans Trias i Pujol, Badalona and Section of Digestive Diseases and Nutrition, University of Illinois at Chicago, IL, USA; Hospital Universitari Germans Trias i Pujol, Badalona and Section of Digestive Diseases and Nutrition, University of Illinois at Chicago, IL, USA; Hospital Universitari Germans Trias i Pujol, Badalona and Section of Digestive Diseases and Nutrition, University of Illinois at Chicago, IL, USA; Hospital Universitari Germans Trias i Pujol, Badalona and Section of Digestive Diseases and Nutrition, University of Illinois at Chicago, IL, USA; Hospital Universitari Germans Trias i Pujol, Badalona and Section of Digestive Diseases and Nutrition, University of Illinois at Chicago, IL, USA; Hospital Universitari Mútua de Terrassa; Hospital Universitari Mútua de Terrassa; Hospital Universitari Mútua de Terrassa; Hospital Universitari Mútua de Terrassa; Hospital Universitari Mútua de Terrassa; Hospital Universitari Mútua de Terrassa; Hospital Universitari Arnau de Vilanova, Lleida; Hospital Universitari Arnau de Vilanova, Lleida; Hospital Universitari Arnau de Vilanova, Lleida; Hospital Universitari Arnau de Vilanova, Lleida; Hospital Universitario de Canarias; Hospital Universitario de Canarias; Hospital Universitario de Canarias; Hospital Universitario de Canarias; Hospital Universitario La Fe, Valencia; Hospital Universitario La Fe, Valencia; Hospital Universitario La Fe, Valencia; Hospital Universitario La Fe, Valencia; Hospital Sant Pau, Barcelona; Hospital Sant Pau, Barcelona; Hospital Sant Pau, Barcelona; Hospital Sant Pau, Barcelona; Hospital Sant Pau, Barcelona; Hospital Sant Pau, Barcelona; Hospital Sant Pau, Barcelona; Hospital Xeral Cies, Vigo; Fundacion Publica Galega de Medicina Xenomica (FPGMX), CIBERER, Genomic Medicine Group-University of Santiago de Compostela, Santiago de Compostela, Galicia, Spain; Fundacion Publica Galega de Medicina Xenomica (FPGMX), CIBERER, Genomic Medicine Group-University of Santiago de Compostela, Santiago de Compostela, Galicia, Spain; Fundacion Publica Galega de Medicina Xenomica (FPGMX), CIBERER, Genomic Medicine Group-University of Santiago de Compostela, Santiago de Compostela, Galicia, Spain; Fundacion Publica Galega de Medicina Xenomica (FPGMX), CIBERER, Genomic Medicine Group-University of Santiago de Compostela, Santiago de Compostela, Galicia, Spain; Hospital Universitario Central de Asturias; Hospital Universitario Central de Asturias; Hospital de Galdácano, Vizcaya; Hospital de Galdácano, Vizcaya; Fundación Hospital de Calahorra (La Rioja) La Rioja; Fundación Hospital de Calahorra (La Rioja) La Rioja; Fundación Hospital de Calahorra (La Rioja) La Rioja; Hospital Royo Villanova, Zaragoza; Hospital Royo Villanova, Zaragoza; Hospital Royo Villanova, Zaragoza; Hospital Royo Villanova, Zaragoza; Hospital Universitario Reina Sofía, Córdoba; 1 Department of Molecular Medicine and Surgery, Karolinska Institute, Stockholm, Sweden; 2 Ludwig Institute for Cancer Research – Stockholm branch, Stockholm, Sweden; 3 Institute of Medical Biometry and Informatics, University Hospital Heidelberg, Heidelberg, Germany; 4 Division of Molecular Genetic Epidemiology, German Cancer Research Center (DKFZ), Heidelberg, Germany; 5 Division of Cancer Epidemiology, German Cancer Research Center (DKFZ), Heidelberg, Germany; 6 Division of Clinical Epidemiology and Aging Research, German Cancer Research Center (DKFZ), Heidelberg, Germany; 7 Galician Public Foundation of Genomic Medicine (FPGMX), Centro de Investigación Biomédica en Red de Enfermedades Raras (CIBERER), Genomics Medicine Group, Hospital Clínico, Santiago de Compostela, University of Santiago de Compostela, Galicia, Spain; 8 Department of Gastroenterology, Hospital Clínic, The Centro de Investigación Biomédica en Red de Enfermedades Hepáticas y Digestivas (CIBERehd), Institut d'Investigacions Biomèdiques August Pi i Sunyer (IDIBAPS), University of Barcelona, Barcelona, Catalonia, Spain; 9 Institute of Experimental Medicine, Academy of Sciences of the Czech Republic, Prague, Czech Republic; 10 First Medical Faculty of the Charles University, Prague, Czech Republic; 11 Division of Clinical Epidemiology and Aging Research, German Cancer Research Center (DKFZ), Heidelberg, Germany; 12 School of Biomedical Science and Pharmacy, University of Newcastle, and the Hunter Medical Research Institute, Newcastle, Australia; 13 School of Science and IT, University of Newcastle, Newcastle, Australia; 14 Unit of Molecular Bases of Genetic Risk and Genetic Testing, Department of Preventive and Predictive Medicine, Fondazione IRCCS Istituto Nazionale dei Tumori, Milan, Italy; 15 Fondazione IFOM, Istituto FIRC di Oncologia Molecolare, Milan Italy; 16 Department of Experimental Oncology and Molecular Medicine, Fondazione IRCCS Istituto Nazionale dei Tumori, Milan, Italy; 17 Department of Family Medicine, Case Center for Transdisciplinary Research on Energetics and Cancer, Case Comprehensive Cancer Center, Case Western Reserve University, Cleveland, Ohio, United States of America; 18 University of Southern California, Norris Comprehensive Cancer Centre, Los Angeles, California, United States of America; 19 Colon Cancer Genetics Group, Institute of Genetics and Molecular Medicine, University of Edinburgh and MRC Human Genetics Unit, Edinburgh, United Kingdom; 20 Oxford NIHR Comprehensive Biomedical Research Centre, Oxford, United Kingdom; 21 IDIBELL-Institut Català d'Oncologia (ICO), CIBER Epidemiología y Salud Pública (CIBERESP) and University of Barcelona, L'Hospitalet de Llobregat, Barcelona, Spain; 22 Department of Pathology, Leiden University Medical Center, Leiden, The Netherlands; 23 Department of Human Genetics and Department of Clinical Genetics, Leiden University Medical Center, Leiden, The Netherlands; 24 Division of Genetics, Hunter Area Pathology Service, John Hunter Hospital, Newcastle, NSW Australia; 25 Department of General Internal Medicine, University Hospital Schleswig-Holstein, Kiel, Germany; 26 Institute for Community Medicine, University Medicine Greifswald, Greifswald, Germany; 27 Department of General and Thoracic Surgery, Christian-Albrechts-University, Kiel, Germany; University of Illinois at Chicago, United States of America

## Abstract

In the last four years, Genome-Wide Association Studies (GWAS) have identified sixteen low-penetrance polymorphisms on fourteen different loci associated with colorectal cancer (CRC). Due to the low risks conferred by known common variants, most of the 35% broad-sense heritability estimated by twin studies remains unexplained. Recently our group performed a case-control study for eight Single Nucleotide Polymorphisms (SNPs) in 4 CRC genes. The present investigation is a follow-up of that study. We have genotyped six SNPs that showed a positive association and carried out a meta-analysis based on eight additional studies comprising in total more than 8000 cases and 6000 controls. The estimated recessive odds ratio for one of the SNPs, rs3219489 (MUTYH Q338H), decreased from 1.52 in the original Swedish study, to 1.18 in the Swedish replication, and to 1.08 in the initial meta-analysis. Since the corresponding summary probability value was 0.06, we decided to retrieve additional information for this polymorphism. The incorporation of six further studies resulted in around 13000 cases and 13000 controls. The newly updated OR was 1.03. The results from the present large, multicenter study illustrate the possibility of decreasing effect sizes with increasing samples sizes. Phenotypic heterogeneity, differential environmental exposures, and population specific linkage disequilibrium patterns may explain the observed difference of genetic effects between Sweden and the other investigated cohorts.

## Introduction

In recent years low-risk common alleles have attracted increasing attention in the search for the “missing heritability” in colorectal cancer (CRC). It concerns the part of heritability that cannot be explained by mutations in already known high-risk genes but should, according to twin studies, account for about 35% [1]. Known high-penetrance germline mutations in CRC genes contribute for less than 6% of the observed cases [2]. Therefore, much of the remaining inherited variation in genetic susceptibility is probably due to multiple low-penetrance variants, both common and rare.

To date sixteen common variants have been identified through large multi-centre genome-wide association studies (GWAS) [3]. Taken together, however, they only explain a small proportion of familial CRC cases. Although the risk associated with each of these variants is modest, they contribute to the disease burden due to their high frequency in the population and the possibility of acting in concert with each other, which may increase the individual's risk of developing CRC [4].

Against this background, a few years ago we attempted to assess the role of eight SNPs in four already known CRC genes (*APC*, *MLH1*, *MSH6* and *MUTYH*) through a case-control association study in the Swedish population [5]. These 8 SNPs had been previously studied, but their pathogenicity was unknown and they were assumed to constitute polymorphisms. In our first study several positive associations were detected but, due to limited sample size (1785 cases and 1722 controls) [5], the results needed to be validated in a follow-up study.

The present study was an initiative of the COGENT consortium [4], [6], where different groups offered to extend the genotyping to other non-Swedish cohorts for SNPs showing statistically significant associations in at least one analysis of the original study. This restricted the analysis to six out of the original eight SNPs.

## Materials and Methods

### Ethics statement

Collection of blood samples and clinical information from patients and controls was obtained with informed consent in accordance with the tenets of the Declaration of Helsinki. All participants gave written informed consent to take part in the study. The study was undertaken in accordance with the Swedish legislation of ethical permission (2003:460) and approved by the Stockholm Regional Research Ethical Committee (Dnr 2002:489).

### Mutation screening

Six SNPs in four different CRC genes were included in the analysis: rs459552:T>A (*APC* D1822V), rs1799977:A>G (*MLH1* I219V), rs1800932:A>G (*MSH6* P92P), rs1800935:T>C (*MSH6* D180D), rs3219484:G>A (*MUTYH* V22M) and rs3219489:G>C (*MUTYH* Q338H). *MUTYH* Q338H corresponds to Q324H in our first study [5]. The SNP nomenclature was modified to meet the Human Genome Variation Society's (HGVS) guidelines, which recommends the use of a reference sequence representing the largest theoretically known transcript. For *MUTYH* this corresponds to NM_001128425.1 and NP_001121897.1 for mRNA and protein, respectively [7], [8], [9].

### Subjects

Details regarding the number of cases and controls in all fourteen studies are summarized in Table S1. One SNP, rs459552 (*APC* D1822V), was genotyped in seven studies, for a total of 8654 cases and 7731 controls. Four SNPs, rs1799977 (*MLH1* I219V), rs1800932 (*MSH6* P92P), rs1800935 (*MSH6* D180D) and rs3219484 (*MUTYH* V22M) were genotyped in 8 studies for a total of 8308 cases and 7434 controls. The SNP with rs number 3219489 (*MUTYH* Q338H) was genotyped in 13 cohorts for a total of 12902 cases and 14602 controls.

For all the subjects genomic DNA was extracted from peripheral blood by standard procedures. Additional information regarding localization of the tumor, age at diagnosis, gender and ethnicity was retrieved whenever possible. Out of 5770 controls with ethnicity information, 5647 were of Caucasian origin, the rest being mostly African American.

### Genotyping

In studies 1, 5, 6, 7, 8, 9 and 10 SNPs were genotyped using the TaqMan SNP Genotyping Assay (Applied Biosystem, Foster City, CA). Genotyping in study 2 and 12 (controls only) was carried out by using the KASPar chemistry of the K-bioscience (Hoddesdon, Herts, UK) (http://www.kbioscience.co.uk/reagents/KASP_manual.pdf), which is a competitive allele-specific PCR SNP genotyping system that uses FRET quencher cassette oligos. Study 3 genotyped with the MassARRAY (Sequenom Inc., San Diego, USA) technology. Study 4 genotyped by means of fluorescent hybridization probe melting curves using the Light Cycler instrument (Roche). Study 11 genotyped using Illumina HumanHap 550 Bead Arrays. Study 12 was genotyped by Sanger sequencing (cases only). Studies 13 and 14 were genotyped using Illumina HumanHap300 and Illumina HumanHap240S.

### Statistical analysis

Deviations of observed genotype frequencies in controls from those expected under Hardy-Weinberg equilibrium were assessed by χ^2^ tests. Risks of CRC associated with genotypes were compared by odds ratios (ORs) with corresponding confidence intervals (CIs) based on logistic regression. Study heterogeneity was summarized using a Mantel–Haenszel test but we assumed that the studies were random samples from a general population and used a random effect model to summarize OR estimates under dominant, recessive and additive penetrance models in the meta-analyses. Results were represented by forest plots as follows: confidence intervals for each individual study were indicated by horizontal lines, single ORs by squares and summary estimates by diamonds with horizontal limits at confidence limits and width inversely proportional to the standard error. Meta-analyses were performed using the package *rmeta* in the free software environment for statistical computing R.

## Results

The distribution of the genotypes in controls did not deviate from Hardy-Weinberg equilibrium in any study. Mantel-Haenszel tests identified study heterogeneity for rs1800932 (*MSH6* P92P) under recessive and additive penetrance, with p-values equal to 0.04 and 0.03, respectively (Table S2). This does not constitute a major issue since this SNP showed no differences between the genotype distributions of cases and controls either in single studies or in the global analysis. Study heterogeneity was not found for any other SNP. Genotyping results for the 6 SNPs based on studies 1–8 are presented in Table S2.

The only SNP that was marginally significant in the meta-analysis was rs3219489 (*MUTYH* Q338H), both under a recessive model (summary OR = 1.08, 95% CI 1.00 to 1.17; p = 0.05) and assuming additive allelic effects (summary OR = 1.07, 95% CI 1.00 to 1.14; p = 0.06). We ascribe the combined result mainly to the Swedish study, with individual ORs of 1.18 (95% CI = 1.01–1.38, recessive model) and 1.19 (95% CI = 1.05–1.35, additive model) (Table S2). The goodness of fit was slightly better for the recessive than for the additive model, and the recessive and additive models clearly outperformed the dominant model.

In an attempt to validate the findings under recessive inheritance, we set up collaborations with additional groups and requested to genotype rs3219489 in their cohorts. In the end, additional 4234 cases and 6800 controls were included, adding up to a total of 12232 cases and 13380 controls (Table S3).

**Figure 1:**
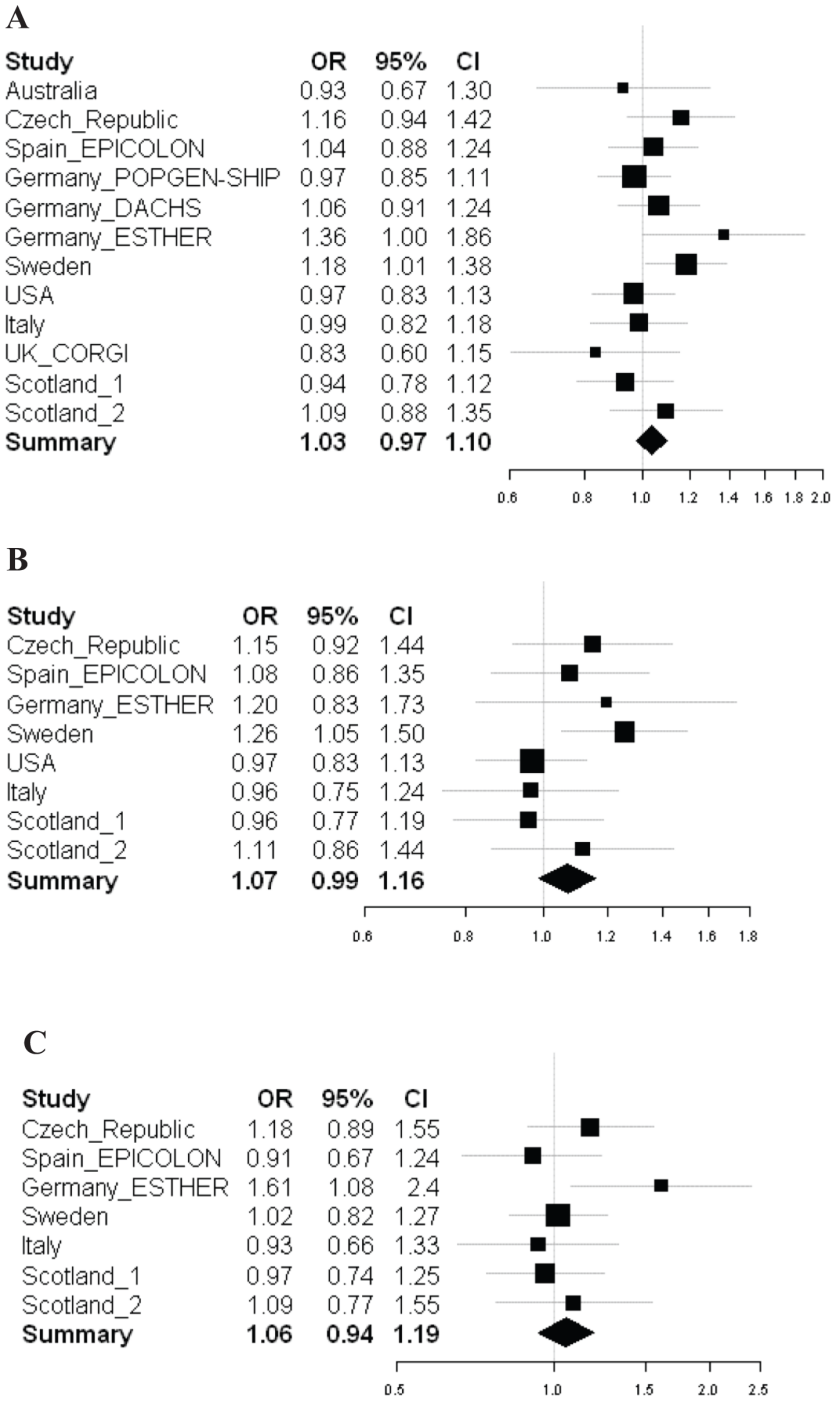
Forest plots with observed odds ratios and 95% confidence intervals for rs3219489 (MUTYH Q338H) under a recessive penetrance model in colorectal cancer (A), colon cancer only (B) and rectal cancer only (C).

We updated the meta-analysis once more considering all samples regardless of tumor localization as well as stratifying them for colon and rectal tumors. As shown in Table S4, data were available for 4573 colon and 1774 rectal cancer cases. Results from the updated meta-analyses are presented in Figure 1. The new summary OR for colorectal cancer was 1.03 (95% CI 0.97 to 1.10, probability value 0.25) (Figure 1A). The summary OR was practically identical after adjustment for age and gender OR = 1.03 (95% CI 0.93 to 1.13). Study heterogeneity was not noticed (P = 0.29, data not shown). The combined OR for colon cancer was 1.07 (95% CI 0.99 to 1.16, probability values 0.09 (OR = 1) and 0.37 (study homogeneity) (Figure 1B) and for rectal cancer was 1.06 (95% CI 0.94 to 1.19, probability values 0.37 (OR = 1) and 0.31 (study homogeneity)) (Figure 1C).

## Discussion

In the present investigation we performed a case-control association study for six out of eight previously investigated SNPs [5]. For five of them, rs459552 (*APC* D1822V), rs1799977 (*MLH1* I219V), rs1800932 (*MSH6* P92P), rs1800935 (*MSH6* D180D) and rs3219484 (*MUTYH* V22M) samples were retrieved from eight additional studies totaling 8308 cases and 7434 controls. For the sixth SNP, rs3219489 (*MUTYH* Q338H), which was selected based on promising results from two samples of Swedish origin (study 8 in the present manuscript and reference [5]), we set up an even larger replication dataset comprising 14 different studies with a total of 12232 cases and 13380 controls.

For all SNPs included in the analysis we were unable to confirm the associations with CRC risk found in the Swedish population. In particular, the recessive ORs of CRC for rs3219489 decreased from 1.52 in the original Swedish study to 1.18 in the Swedish replication cohort, to 1.08 (95% CI 1.00 to 1.17) in the first meta-analysis and to 1.03 (95% CI 0.97 to 1.10) in the updated meta-analysis (Table S2). The summary ORs in the extended meta-analyses were 1.07 (95% CI 0.99 to 1.16) for colon cancer and 1.06 (95% CI 0.94 to 1.19) for rectal cancer, in contrast with results based on Swedish samples. The updated meta-analysis had statistical power of 99% to detect a recessive OR of 1.52 and a power of 89% to detect a recessive OR of 1.18 (Type I error rate 5% and prevalence of CC genotypes among controls 5.6%). Biological plausibility was also existent. MUTYH Q338H is interesting because it represents a missense change in the MUTYH protein, which is involved in the base excision repair (BER) pathway. A common product of oxidative damage to 2′-deoxyguanosine is 7,8-dihydro-8-oxo-2′-deoxyguanosine (OG) [10], [11]. In mammalian cells OG has been shown to be highly mutagenic and leading to an increased rate of G→T transversions, due to its miscoding properties that cause a mispairing with an adenine during DNA replication to form a stable OG:A mismatch [11], [12]. The BER pathway plays an important role in repairing this type of DNA damage through the action of the mutY homolog *MUTYH*, in concert with *OGG1* and *MTH1* [11], [13]. It is well established that biallelic mutations in *MUTYH* gene introduce G:C to T:A transversions also in the adenomatous polyposis coli (APC) gene, leading to genomic instability and abnormal and dis-regulated cell proliferation in the colonic epithelium [14], [15]. Patients with two mutations in the *MUTYH* gene develop the *MUTYH*-associated polyposis (MAP) syndrome [13].

To date, 85 different MAP-associated mutations have been found [16], scattered throughout the entire length of the protein, but only 3 (including Q338H) map within putative protein interaction domains as revealed by the recently solved crystal structure of hMUTYH [17]. It is tempting to speculate that Q338H might affect this protein-protein interaction, but additional experimental support is warranted.

The contrasting results on rs3219489 and its association with CRC risk in the Swedish versus other populations might suggest that the effect of this variant is specific for the Swedish population or not large enough in the other populations to be detected with the present sample size. For example, the statistical power of the updated meta-analysis was only 43% to detect a recessive OR of 1.10 (Type I error rate 5% and prevalence of CC genotypes among controls 5.6%). A closer look at the data actually shows that one of the German cohorts (ESTHER) gave results in agreement with our Swedish cohorts, with OR = 1.36 (95% CI 1.00 to 1.86) for colorectal cancer (Figure 1A) and OR = 1.61 (95% CI 1.08 to 2.40) for rectal cancer (Figure 1C). This is likely a spurious result due to the small size of that cohort (318 cases and 365 controls).

On the other hand, in agreement with Swedish results, rs3219489 has also been shown to be associated with CRC risk in three independent studies in the Japanese population [18], [19], [20] and among African-Americans (Yuan et al., 2nd InSiGHT meeting, Yokohama, Japan, unpublished) even though all these studies have a limited sample size and the results need further validation.

It is also possible that rs3219489 represents a risk-associated variant in the Swedish population in combination with environmental factors in the broad sense. For example, screening programs for CRC in Sweden could result in a diagnosis earlier in life, thus inflating the ORs estimated in Sweden. Another alternative is that the polymorphism is in linkage disequilibrium with other unidentified causal variants. The marker and the causal variant could be located on the same risk haplotype in the Swedish population and on different haplotypes in other populations.

Independently of the unknown reason for replication failure, the results from the present study clearly illustrate the possibility of decreasing effect sizes with increasing collections of individuals, a phenomenon well-known in the field of genetic epidemiology denominated the winner's curse [21]. It should be kept in mind that this outcome is rather expected in association studies, in particular those dealing with regionally heterogeneous complex diseases.

## Supporting Information

Table S1 **Number of cases and controls genotyped in the fourteen studies.**
(DOC)

Table S2 **Genotype counts and allele frequencies for rs459552 (APC D1822V), rs1799977 (MLH I219V), rs1800932 (MSH6 P92P), rs1800935 (MSH6 D180D), rs3219484 (MUTYH V22M) and rs3219489 (MUTYH Q338H).** The estimated odds ratios with 95% confidence intervals for individual studies are also shown, together with combined ORs, 95% CIs and probability values for OR = 1 based on random effects model and probability values for study homogeneity under dominant, additive and recessive penetrance.
(DOC)

Table S3 **Genotype counts and allele frequencies for rs3219489 (MUTYH Q338H).**
(DOC)

Table S4 **Genotype counts for colon and rectal cancer cases in studies with available information on tumor location.**
(DOC)
