# Supplementary material for: Meta-Analysis of Mismatch Repair Polymorphisms within the Cogent Consortium for Colorectal Cancer Susceptibility
Source: PLoS One. 2013 Sep 6;8(9):e72091. doi: 10.1371/journal.pone.0072091 (PMC3765450; doi:10.1371/journal.pone.0072091)
Supplement: Table S1 — Number of cases and controls genotyped in the fourteen studies. (DOC) [file pone.0072091.s001.doc]

**Table S1**

| **Study** | | **No. cases** | **No. controls** | **Investigated SNPs** |
| --- | --- | --- | --- | --- |
| 1 | Australia | 350 | 352 | All six |
| 2 | Czech Republic | 990 | 679 | All six |
| 3 | Spain_EPICOLON | 1389 | 976 | All six |
| 4 | Spain_2 | 346 | 297 | rs459552 |
| 5 | Germany_POPGEN-SHIP | 2431 | 2200 | All six |
| 6 | Germany_DACHS | 1373 | 1480 | All six |
| 7 | Germany_ESTHER | 341 | 368 | All six |
| 8 | Sweden | 1434 | 1379 | All six |
| 9 | USA | 1073 | 1714 | rs3219489 |
| 10 | Italy | 622 | 2574 | rs3219489 |
| 11 | UK_CORGI | 681 | 200 | rs3219489 |
| 12 | The Netherlands | 288 | 589 | rs3219489 |
| 13 | Scotland_1 | 960 | 985 | rs3219489 |
| 14 | Scotland_2 | 624 | 809 | rs3219489 |
|  | **Total** | **12902** | **14 602** |  |
