# Supplementary material for: Meta-Analysis of Mismatch Repair Polymorphisms within the Cogent Consortium for Colorectal Cancer Susceptibility
Source: PLoS One. 2013 Sep 6;8(9):e72091. doi: 10.1371/journal.pone.0072091 (PMC3765450; doi:10.1371/journal.pone.0072091)
Supplement: Table S2 — Genotype counts and allele frequencies for rs459552 (APC D1822V), rs1799977 (MLH I219V), rs1800932 (MSH6 P92P), rs1800935 (MSH6 D180D), rs3219484 (MUTYH V22M) and rs3219489 (MUTYH Q338H). The estimated odds ratios with 95% confidence intervals for individual studies are also shown, together with combined ORs, 95% CIs and probability values for OR = 1 based on random effects model and probability values for study homogeneity under dominant, additive and recessive penetrance. (DOC) [file pone.0072091.s002.doc]

**Table S2**

| **Study** | | **Tot.**  **cases** | **Case genotypes** | | | **Tot.**  **controls** | **Control genotypes** | | | **MAF**  **cases** | **MAF**  **controls** | **Dominant** | | | | **Additive** | | | | **Recessive** | | | |
| --- | --- | --- | --- | --- | --- | --- | --- | --- | --- | --- | --- | --- | --- | --- | --- | --- | --- | --- | --- | --- | --- | --- | --- |
|  |  |  | **TT** | **TA** | **AA** |  | **TT** | **TA** | **AA** |  |  | **OR** | **95% CI** | **P_OR_** | **P_Het_** | **OR** | **95% CI** | **P_OR_** | **P_Het_** | **OR** | **95% CI** | **P_OR_** | **P_Het_** |
| *rs459552:T>A (APC D1822V)* | |  |  |  |  |  |  |  |  |  |  |  |  |  |  |  |  |  |  |  |  |  |  |
| 1 | Australia | 327 | 197 | 119 | 111 | 344 | 205 | 118 | 21 | 0.784 | 0.767 | 1.87 | 0.87-4.00 |  |  | 1.10 | 0.85-1.43 |  |  | 1.03 | 0.75-1.41 |  |  |
| 2 | Czech Republic | 972 | 524 | 367 | 81 | 661 | 346 | 275 | 40 | 0.728 | 0.731 | 0.71 | 0.47-1.06 |  |  | 0.98 | 0.84-1.15 |  |  | 1.06 | 0.87-1.30 |  |  |
| 3 | Spain_EPICOLON | 1385 | 861 | 466 | 58 | 974 | 600 | 330 | 44 | 0.790 | 0.785 | 1.08 | 0.72-1.63 |  |  | 1.03 | 0.89-1.19 |  |  | 1.02 | 0.86-1.22 |  |  |
| 4 | Spain_2 | 346 | 224 | 108 | 14 | 297 | 197 | 84 | 16 | 0.803 | 0.805 | 1.35 | 0.64-2.86 |  |  | 0.99 | 0.75-1.32 |  |  | 0.93 | 0.67-1.30 |  |  |
| 5 | Germany_POPGEN-SHIP | 2384 | 1406 | 835 | 143 | 1459 | 822 | 540 | 97 | 0.765 | 0.748 | 1.12 | 0.85-1.46 |  |  | 1.09 | 0.98-1.22 |  |  | 1.11 | 0.97-1.27 |  |  |
| 6 | Germany_DACHS | 1307 | 785 | 449 | 73 | 1429 | 865 | 487 | 77 | 0.772 | 0.776 | 0.96 | 0.69-1.35 |  |  | 0.98 | 0.86-1.12 |  |  | 0.98 | 0.84-1.15 |  |  |
| 7 | Germany_ESTHER | 341 | 195 | 131 | 15 | 363 | 234 | 105 | 24 | 0.764 | 0.789 | 1.54 | 0.78-3.03 |  |  | 0.86 | 0.67-1.12 |  |  | 0.74 | 0.54-1.00 |  |  |
| 8 | Sweden | 1385 | 830 | 480 | 75 | 1346 | 787 | 499 | 60 | 0.773 | 0.770 | 0.81 | 0.57-1.16 |  |  | 1.01 | 0.89-1.15 |  |  | 1.06 | 0.91-1.24 |  |  |
|  | **Total** | 8447 | 5022 | 2955 | 470 | 6873 | 4056 | 2438 | 379 | 0.769 | 0.767 | 1.02 | 0.85-1.23 | 0.82 | 0.18 | 1.02 | 0.97-1.08 | 0.45 | 0.75 | 1.03 | 0.96-1.10 | 0.40 | 0.42 |
|  |  |  |  |  |  |  |  |  |  |  |  |  |  |  |  |  |  |  |  |  |  |  |  |
| *rs1799977:A>G (MLH1 I219V)* | |  | **AA** | **AG** | **GG** |  | **AA** | **AG** | **GG** |  |  |  |  |  |  |  |  |  |  |  |  |  |  |
| 1 | Australia | 321 | 158 | 135 | 28 | 342 | 167 | 137 | 38 | 0.702 | 0.689 | 1.31 | 0.77-2.21 |  |  | 1.07 | 0.84-1.36 |  |  | 1.02 | 0.74-1.39 |  |  |
| 2 | Czech Republic | 974 | 437 | 443 | 94 | 662 | 285 | 305 | 72 | 0.676 | 0.661 | 1.14 | 0.82-1.59 |  |  | 1.07 | 0.92-1.25 |  |  | 1.08 | 0.88-1.32 |  |  |
| 3 | Spain_EPICOLON | 1389 | 602 | 622 | 165 | 976 | 455 | 403 | 118 | 0.657 | 0.673 | 1.02 | 0.79-1.32 |  |  | 0.93 | 0.82-1.06 |  |  | 0.88 | 0.74-1.04 |  |  |
| 5 | Germany_POPGEN-SHIP | 2390 | 1096 | 1036 | 258 | 1460 | 660 | 633 | 167 | 0.675 | 0.669 | 1.07 | 0.86-1.32 |  |  | 1.03 | 0.93-1.14 |  |  | 1.03 | 0.90-1.17 |  |  |
| 6 | Germany_DACHS | 1296 | 625 | 553 | 118 | 1417 | 622 | 642 | 153 | 0.696 | 0.665 | 1.21 | 0.93-1.56 |  |  | 1.15 | 1.02-1.29 |  |  | 1.19 | 1.02-1.39 |  |  |
| 7 | Germany_ESTHER | 341 | 145 | 157 | 39 | 368 | 159 | 166 | 43 | 0.655 | 0.658 | 1.02 | 0.64-1.64 |  |  | 0.99 | 0.79-1.24 |  |  | 0.97 | 0.72-1.32 |  |  |
| 8 | Sweden | 1357 | 655 | 568 | 134 | 1343 | 628 | 582 | 133 | 0.692 | 0.684 | 1.00 | 0.78-1.30 |  |  | 1.04 | 0.92-1.17 |  |  | 1.06 | 0.91-1.24 |  |  |
|  | **Total** | 8068 | 3718 | 3514 | 836 | 6568 | 2976 | 2868 | 724 | 0.678 | 0.671 | 1.09 | 0.97-1.21 | 0.13 | 0.92 | 1.04 | 0.99-1.10 | 0.14 | 0.39 | 1.04 | 0.96-1.12 | 0.36 | 0.26 |
|  |  |  |  |  |  |  |  |  |  |  |  |  |  |  |  |  |  |  |  |  |  |  |  |
| *rs1800932:A>G (MSH6 P92P)* | |  | **AA** | **AG** | **GG** |  | **AA** | **AG** | **GG** |  |  |  |  |  |  |  |  |  |  |  |  |  |  |
| 1 | Australia | 336 | 200 | 121 | 15 | 347 | 233 | 107 | 7 | 0.775 | 0.826 | 0.44 | 0.17-1.12 |  |  | 0.73 | 0.55-0.96 |  |  | 0.72 | 0.52-0.99 |  |  |
| 2 | Czech Republic | 967 | 661 | 278 | 28 | 665 | 464 | 177 | 24 | 0.827 | 0.831 | 1.26 | 0.71-2.21 |  |  | 0.98 | 0.81-1.18 |  |  | 0.94 | 0.75-1.15 |  |  |
| 3 | Spain_EPICOLON | 1389 | 869 | 458 | 62 | 976 | 567 | 362 | 47 | 0.790 | 0.766 | 1.08 | 0.73-1.61 |  |  | 1.15 | 1.00-1.33 |  |  | 1.21 | 1.02-1.43 |  |  |
| 5 | Germany_POPGEN-SHIP | 2390 | 1637 | 686 | 67 | 1461 | 1009 | 410 | 42 | 0.828 | 0.831 | 1.03 | 0.69-1.53 |  |  | 0.98 | 0.87-1.11 |  |  | 0.97 | 0.84-1.12 |  |  |
| 6 | Germany_DACHS | 1318 | 864 | 410 | 44 | 1433 | 948 | 416 | 69 | 0.811 | 0.807 | 1.46 | 0.99-2.17 |  |  | 1.03 | 0.90-1.18 |  |  | 0.97 | 0.83-1.14 |  |  |
| 7 | Germany_ESTHER | 341 | 221 | 106 | 14 | 368 | 240 | 110 | 18 | 0.804 | 0.802 | 1.20 | 0.58-2.49 |  |  | 1.01 | 0.77-1.32 |  |  | 0.98 | 0.72-1.35 |  |  |
| 8 | Sweden | 1408 | 1025 | 354 | 29 | 1363 | 943 | 377 | 43 | 0.854 | 0.830 | 1.55 | 0.95-2.52 |  |  | 1.19 | 1.03-1.38 |  |  | 1.19 | 1.01-1.41 |  |  |
|  | **Total** | 8149 | 5477 | 2413 | 259 | 6613 | 4404 | 1959 | 250 | 0.820 | 0.814 | 1.18 | 0.95-1.46 | 0.12 | 0.26 | 1.03 | 0.93-1.13 | 0.60 | 0.04 | 1.01 | 0.91-1.13 | 0.82 | 0.04 |

**Table S2 (Cont.)**

| **Study** | | **Tot.**  **cases** | **Case genotypes** | | | **Tot.**  **controls** | **Control genotypes** | | | **MAF**  **cases** | **MAF**  **controls** | **Dominant** | | | | **Additive** | | | | **Recessive** | | | |
| --- | --- | --- | --- | --- | --- | --- | --- | --- | --- | --- | --- | --- | --- | --- | --- | --- | --- | --- | --- | --- | --- | --- | --- |
|  |  |  | **TT** | **TA** | **AA** |  | **TT** | **TA** | **AA** |  |  | **OR** | **95% CI** | **P_OR_** | **P_Het_** | **OR** | **95% CI** | **P_OR_** | **P_Het_** | **OR** | **95% CI** | **P_OR_** | **P_Het_** |
| *rs1800935:T>C (MSH6 D180D)* | |  |  |  |  |  |  |  |  |  |  |  |  |  |  |  |  |  |  |  |  |  |  |
| 1 | Australia | 333 | 147 | 151 | 35 | 352 | 168 | 164 | 20 | 0.668 | 0.710 | 0.51 | 0.29-0.92 |  |  | 0.82 | 0.65-1.04 |  |  | 0.87 | 0.64-1.18 |  |  |
| 2 | Czech Republic | 985 | 454 | 440 | 91 | 672 | 328 | 281 | 63 | 0.684 | 0.697 | 1.02 | 0.72-1.43 |  |  | 0.94 | 0.81-1.10 |  |  | 0.90 | 0.73-1.10 |  |  |
| 3 | Spain_EPICOLON | 1388 | 668 | 573 | 147 | 976 | 451 | 436 | 99 | 0.688 | 0.685 | 0.95 | 0.72-1.25 |  |  | 1.03 | 0.91-1.17 |  |  | 1.08 | 0.91-1.28 |  |  |
| 5 | Germany_POPGEN-SHIP | 2379 | 1205 | 971 | 203 | 1212 | 623 | 478 | 111 | 0.711 | 0.711 | 1.08 | 0.84-1.38 |  |  | 1.00 | 0.89-1.11 |  |  | 0.97 | 0.84-1.12 |  |  |
| 6 | Germany_DACHS | 1299 | 634 | 551 | 114 | 1432 | 720 | 563 | 149 | 0.700 | 0.699 | 1.21 | 0.93-1.57 |  |  | 1.00 | 0.89-1.13 |  |  | 0.94 | 0.81-1.10 |  |  |
| 7 | Germany_ESTHER | 341 | 165 | 142 | 34 | 367 | 174 | 154 | 39 | 0.692 | 0.684 | 1.07 | 0.65-1.76 |  |  | 1.04 | 0.83-1.31 |  |  | 1.04 | 0.77-1.41 |  |  |
| 8 | Sweden | 1416 | 753 | 546 | 117 | 1361 | 661 | 583 | 117 | 0.725 | 0.700 | 1.04 | 0.79-1.37 |  |  | 1.13 | 1.00-1.27 |  |  | 1.20 | 1.03-1.40 |  |  |
|  | **Total** | 8141 | 4026 | 3374 | 741 | 6372 | 3125 | 2649 | 598 | 0.702 | 0.698 | 1.02 | 0.89-1.17 | 0.72 | 0.26 | 1.01 | 0.95-1.07 | 0.71 | 0.26 | 1.01 | 0.92-1.10 | 0.85 | 0.15 |
|  |  |  |  |  |  |  |  |  |  |  |  |  |  |  |  |  |  |  |  |  |  |  |  |
| *rs3219484:G>A (MUTYH V22M)* | |  | **GG** | **GA** | **AA** |  | **GG** | **GA** | **AA** |  |  |  |  |  |  |  |  |  |  |  |  |  |  |
| 1 | Australia | 343 | 294 | 48 | 1 | 352 | 297 | 53 | 2 | 0.927 | 0.919 | 1.95 | 0.17-22.74 |  |  | 1.12 | 0.75-1.68 |  |  | 1.11 | 0.73-1.70 |  |  |
| 2 | Czech Republic | 969 | 817 | 146 | 6 | 671 | 577 | 91 | 3 | 0.918 | 0.928 | 0.72 | 0.17-2.98 |  |  | 0.88 | 0.67-1.15 |  |  | 0.88 | 0.66-1.16 |  |  |
| 3 | Spain_EPICOLON | 1388 | 1268 | 118 | 2 | 975 | 888 | 81 | 6 | 0.956 | 0.952 | 4.29 | 0.84-22.01 |  |  | 1.09 | 0.82-1.44 |  |  | 1.04 | 0.77-1.39 |  |  |
| 5 | Germany_POPGEN-SHIP | 2404 | 2030 | 359 | 15 | 1464 | 1257 | 200 | 7 | 0.919 | 0.927 | 0.77 | 0.31-1.92 |  |  | 0.90 | 0.75-1.07 |  |  | 0.89 | 0.74-1.08 |  |  |
| 6 | Germany_DACHS | 1331 | 1175 | 151 | 5 | 1445 | 1302 | 136 | 7 | 0.940 | 0.948 | 1.29 | 0.40-4.17 |  |  | 0.85 | 0.67-1.07 |  |  | 0.83 | 0.65-1.06 |  |  |
| 7 | Germany_ESTHER | 341 | 292 | 47 | 2 | 368 | 317 | 48 | 3 | 0.925 | 0.927 | 1.39 | 0.22-8.70 |  |  | 0.98 | 0.65-1.47 |  |  | 0.96 | 0.62-1.48 |  |  |
| 8 | Sweden | 1399 | 1184 | 208 | 7 | 1358 | 1142 | 206 | 10 | 0.921 | 0.917 | 1.48 | 0.55-3.96 |  |  | 1.05 | 0.86-1.28 |  |  | 1.04 | 0.85-1.04 |  |  |
|  | **Total** | 8175 | 7060 | 1077 | 38 | 6633 | 5780 | 815 | 38 | 0.929 | 0.933 | 1.22 | 0.75-1.98 | 0.41 | 0.64 | 0.95 | 0.87-1.05 | 0.32 | 0.62 | 0.94 | 0.85-1.04 | 0.21 | 0.73 |
|  |  |  |  |  |  |  |  |  |  |  |  |  |  |  |  |  |  |  |  |  |  |  |  |
| *rs3219489:G>C (MUTYH Q338H)* | |  | **GG** | **GC** | **CC** |  | **GG** | **GC** | **CC** |  |  |  |  |  |  |  |  |  |  |  |  |  |  |
| 1 | Australia | 337 | 203 | 111 | 23 | 340 | 199 | 119 | 22 | 0.767 | 0.760 | 1.17 | 0.62-2.22 |  |  | 0.98 | 0.75-1.29 |  |  | 0.93 | 0.67-1.30 |  |  |
| 2 | Czech Republic | 979 | 586 | 348 | 45 | 676 | 428 | 214 | 34 | 0.776 | 0.791 | 0.91 | 0.57-1.45 |  |  | 1.09 | 0.92-1.30 |  |  | 1.16 | 0.94-1.42 |  |  |
| 3 | Spain_EPICOLON | 1305 | 650 | 554 | 101 | 952 | 484 | 400 | 68 | 0.710 | 0.718 | 1.09 | 0.79-1.51 |  |  | 1.04 | 0.91-1.19 |  |  | 1.04 | 0.88-1.24 |  |  |
| 5 | Germany_POPGEN-SHIP | 2364 | 1465 | 794 | 105 | 1461 | 895 | 499 | 67 | 0.788 | 0.783 | 0.97 | 0.70-1.33 |  |  | 0.97 | 0.87-1.09 |  |  | 0.97 | 0.85-1.11 |  |  |
| 6 | Germany_DACHS | 1304 | 772 | 458 | 74 | 1429 | 867 | 478 | 84 | 0.768 | 0.774 | 0.96 | 0.69-1.34 |  |  | 1.04 | 0.91-1.18 |  |  | 1.06 | 0.91-1.24 |  |  |
| 7 | Germany_ESTHER | 318 | 162 | 134 | 22 | 365 | 214 | 130 | 21 | 0.720 | 0.764 | 1.22 | 0.65-2.29 |  |  | 1.26 | 0.98-1.62 |  |  | 1.36 | 1.00-1.86 |  |  |
| 8 | Sweden | 1391 | 783 | 509 | 99 | 1357 | 818 | 474 | 65 | 0.746 | 0.777 | 1.52 | 1.10-2.12 |  |  | **1.19** | **1.05-1.35** |  |  | **1.18** | **1.01-1.38** |  |  |
|  | **Total** | 7998 | 4621 | 2908 | 469 | 6580 | 3905 | 2314 | 361 | 0.760 | 0.769 | 1.09 | 0.95-1.26 | 0.22 | 0.42 | 1.07 | 1.00-1.14 | 0.06 | 0.22 | 1.08 | 1.00-1.17 | 0.06 | 0.27 |
