# Supplementary material for: Meta-Analysis of Mismatch Repair Polymorphisms within the Cogent Consortium for Colorectal Cancer Susceptibility
Source: PLoS One. 2013 Sep 6;8(9):e72091. doi: 10.1371/journal.pone.0072091 (PMC3765450; doi:10.1371/journal.pone.0072091)
Supplement: Table S3 — Genotype counts and allele frequencies for rs3219489 (MUTYH Q338H). (DOC) [file pone.0072091.s003.doc]

**Table S3**

| **Study** | | **Tot.**  **cases** | **Case genotypes** | | | **Tot.**  **controls** | **Controls genotypes** | | | **MAF**  **cases** | **MAF**  **controls** | **Dominant** | | | | **Additive** | | | | **Recessive** | | | |
| --- | --- | --- | --- | --- | --- | --- | --- | --- | --- | --- | --- | --- | --- | --- | --- | --- | --- | --- | --- | --- | --- | --- | --- |
|  |  |  | **GG** | **GC** | **CC** |  | **GG** | **GC** | **CC** |  |  | **OR** | **95% CI** | **P_OR_** | **P_Het_** | **OR** | **95% CI** | **P_OR_** | **P_Het_** | **OR** | **95% CI** | **P_OR_** | **P_Het_** |
| 1 | Australia | 337 | 203 | 111 | 23 | 340 | 199 | 119 | 22 | 0.767 | 0.760 | 1.22 | 0.65-2.29 |  |  | 0.98 | 0.75-1.29 |  |  | 0.93 | 0.67-1.30 |  |  |
| 2 | Czech Republic | 979 | 586 | 348 | 45 | 676 | 428 | 214 | 34 | 0.776 | 0.791 | 0.91 | 0.57-1.45 |  |  | 1.09 | 0.92-1.30 |  |  | 1.16 | 0.94-1.42 |  |  |
| 3 | Spain_EPICOLON | 1305 | 650 | 554 | 101 | 952 | 484 | 400 | 68 | 0.710 | 0.718 | 1.09 | 0.79-1.51 |  |  | 1.04 | 0.91-1.19 |  |  | 1.04 | 0.88-1.24 |  |  |
| 5 | Germany_POPGEN-SHIP | 2364 | 1465 | 794 | 105 | 1461 | 895 | 499 | 67 | 0.788 | 0.783 | 0.97 | 0.70-1.33 |  |  | 0.97 | 0.87-1.09 |  |  | 0.97 | 0.85-1.11 |  |  |
| 6 | Germany_DACHS | 1304 | 772 | 458 | 74 | 1429 | 867 | 478 | 84 | 0.768 | 0.774 | 0.96 | 0.69-1.34 |  |  | 1.04 | 0.91-1.18 |  |  | 1.06 | 0.91-1.24 |  |  |
| 7 | Germany_ESTHER | 318 | 162 | 134 | 22 | 365 | 214 | 130 | 21 | 0.720 | 0.764 | 1.22 | 0.65-2.29 |  |  | 1.26 | 0.98-1.62 |  |  | 1.36 | 1.00-1.86 |  |  |
| 8 | Sweden | 1391 | 783 | 509 | 99 | 1357 | 818 | 474 | 65 | 0.746 | 0.777 | 1.52 | 1.10-2.12 |  |  | 1.19 | 1.05-1.35 |  |  | 1.18 | 1.01-1.38 |  |  |
| 9 | USA | 1073 | 595 | 423 | 55 | 1714 | 936 | 676 | 102 | 0.752 | 0.743 | 0.85 | 0.61-1.20 |  |  | 0.96 | 0.84-1.09 |  |  | 0.97 | 0.83-1.13 |  |  |
| 10 | Italy | 610 | 366 | 199 | 45 | 2508 | 1496 | 886 | 126 | 0.763 | 0.773 | 1.51 | 1.05-2.16 |  |  | 1.06 | 0.91-1.23 |  |  | 0.99 | 0.82-1.18 |  |  |
| 11 | UK_CORGI | 681 | 395 | 237 | 49 | 200 | 107 | 78 | 15 | 0.754 | 0.730 | 0.96 | 0.52-1.77 |  |  | 0.88 | 0.68-1.14 |  |  | 0.83 | 0.60-1.15 |  |  |
| 12 | The Netherlands | 288 | 168 | 110 | 10 | 589 | 348 | 211 | 30 | 0.774 | 0.770 | 1.08 | 0.80-1.45 |  |  | 1.03 | 0.78-1.37 |  |  | 0.69 | 0.33-1.45 |  |  |
| 13 | Scotland_1 | 960 | 554 | 355 | 51 | 985 | 547 | 380 | 58 | 0.762 | 0.748 | 0.93 | 0.63-1.37 |  |  | 0.95 | 0.82-1.10 |  |  | 0.94 | 0.78-1.12 |  |  |
| 14 | Scotland_2 | 622 | 315 | 267 | 40 | 804 | 425 | 325 | 54 | 0.721 | 0.731 | 0.95 | 0.62-1.47 |  |  | 1.05 | 0.89-1.24 |  |  | 1.09 | 0.88-1.35 |  |  |
|  | **Total** | 12232 | 7014 | 4499 | 719 | 13380 | 7764 | 4870 | 746 | 0.757 | 0.762 | 1.07 | 0.95-1.21 | 0.26 | 0.33 | 1.03 | 0.98-1.09 | 0.19 | 0.24 | 1.03 | 0.97-1.10 | 0.25 | 0.29 |
