# Supplementary material for: Meta-Analysis of Mismatch Repair Polymorphisms within the Cogent Consortium for Colorectal Cancer Susceptibility
Source: PLoS One. 2013 Sep 6;8(9):e72091. doi: 10.1371/journal.pone.0072091 (PMC3765450; doi:10.1371/journal.pone.0072091)
Supplement: Table S4 — Genotype counts for colon and rectal cancer cases in studies with available information on tumor location. (DOC) [file pone.0072091.s004.doc]

**Table S4**

| **Study** | | **Colon cancer**  **cases** | **Genotype** | | | **Rectal cancer**  **cases** | **Genotype** | | |
| --- | --- | --- | --- | --- | --- | --- | --- | --- | --- |
|  |  |  | **GG** | **GC** | **CC** |  | **GG** | **GC** | **CC** |
| 2 | Czech Republic | 657 | 396 | 230 | 31 | 324 | 193 | 117 | 14 |
| 3 | Spain_EPICOLON | 484 | 237 | 214 | 33 | 203 | 108 | 82 | 13 |
| 7 | Germany_ESTHER | 177 | 96 | 67 | 14 | 141 | 67 | 66 | 8 |
| 8 | Sweden | 822 | 450 | 319 | 53 | 485 | 290 | 156 | 39 |
| 9 | USA | 1073 | 595 | 423 | 55 | - | - | - | - |
| 10 | Italy | 468 | 279 | 157 | 32 | 142 | 87 | 42 | 13 |
| 13 | Scotland_1 | 551 | 312 | 211 | 28 | 319 | 180 | 115 | 24 |
| 14 | Scotland_2 | 341 | 171 | 146 | 24 | 160 | 81 | 73 | 6 |
|  | **Total** | 4573 | 2536 | 1767 | 270 | 1774 | 1006 | 651 | 117 |
